# New Strain of Simian Immunodeficiency Virus Identified in Wild-Born Chimpanzees from Central Africa

**DOI:** 10.1371/journal.pone.0044298

**Published:** 2012-09-12

**Authors:** Sandrine Souquière, Maria Makuwa, Bettina Sallé, Mirdad Kazanji

**Affiliations:** 1 Unité de Rétrovirologie, Centre International de Recherches Médicales de Franceville (CIRMF), Franceville, Gabon; 2 Centre de Primatologie, Centre International de Recherches Médicales de Franceville (CIRMF), BP 769, Franceville, Gabon; 3 Institut Pasteur de Bangui, Réseau International des Instituts Pasteur, Bangui, Central African Republic; The University of Hong Kong, Hong Kong

## Abstract

Studies of primate lentiviruses continue to provide information about the evolution of simian immunodeficiency viruses (SIVs) and the origin and emergence of HIV since chimpanzees in west–central Africa (*Pan troglodytes troglodytes*) were recognized as the reservoir of SIVcpz*Ptt* viruses, which have been related phylogenetically to HIV-1. Using in-house peptide ELISAs to study SIV prevalence, we tested 104 wild-born captive chimpanzees from Gabon and Congo. We identified two new cases of SIVcpz infection in Gabon and characterized a new SIVcpz strain, SIVcpz*Ptt*-Gab4. The complete sequence (9093 bp) was obtained by a PCR-based ‘genome walking’ approach to generate 17 overlapping fragments. Phylogenetic analyses of separated genes (*gag, pol-vif* and *env-nef*) showed that SIVcpz*Ptt*-Gab4 is closely related to SIVcpz*Ptt*-Gab1 and SIVcpz*Ptt*-Gab2. No significant variation in viral load was observed during 3 years of follow-up, but a significantly lower CD4+ T cells count was found in infected than in uninfected chimpanzees (*p*<0.05). No clinical symptoms of SIV infection were observed in the SIV-positive chimpanzees. Further field studies with non-invasive methods are needed to determine the prevalence, geographic distribution, species association, and natural history of SIVcpz strains in the chimpanzee habitat in Gabon.

## Introduction

Simian immunodeficiency virus (SIV), a member of the *Lentivirus* genus (Retroviridae), has been isolated from various African nonhuman primates, including *Cercopithecidae* species and great apes (*Pan troglodytes* and *Gorilla* spp.) [1], [2]. SIV has been clustered into six distinct lineages [3]. SIV from chimpanzees (SIVcpz) has been found to be genetically related to human immunodeficiency virus (HIV-1), with the same genomic organization [4], and the strong homology suggested that HIV-1 originated from chimpanzees [5]. This hypothesis was strengthened by the identification and characterization of two SIVcpz strains (SIVcpz*Ptt*-Gab1 and SIVcpz*Ptt*-Gab2) from captive wild-born chimpanzees in Gabon [6]–[9] and of one strain (SIVcpz*Ptt*-US) in chimpanzees from an unknown central African country but kept in captivity in the USA [4]. Another strain (SIVcpz-Ant), which was not related to the HIV-1 group M (pandemic), N or O (nonpandemic), was characterized in a wild-born captured chimpanzee in Belgium [10].

Studies of hundreds of captive wild-born *P. troglodytes troglodytes (P.t.t)*, revealed four SIVcpzPtt strains (SIVcpz*Ptt*-Cam3, Cam5, Cam13 and Cam 155) in Cameroon [11]–[14]. These SIVcpz sequences clustered with SIVcpz*Ptt*-US and SIVcpz*Ptt*-Gab1. In 1998, identification of the HIV-1 group N strain which does not belong to HIV-1 group M or O, also in Cameroon, provided evidence of a close phylogenetic relation with SIVcpz*Ptt* circulating in the same geographic area [11], [12], [15].

Analysis of faecal samples from wild-living gorillas (*Gorilla gorilla*) in Cameroon showed the presence of a new SIV lineage. These SIVgor viruses, forming a monophyletic lineage within the SIVcpzPtt group, suggested that SIVgor resulted from a chimpanzee-to-gorilla transmission [16]. Although there have been a few cases of SIV infection among western lowland gorillas (*Gorilla gorilla gorilla*), phylogenetic analyses of these SIVgor strains showed their close relation to human HIV-1 group O viruses [16]–[18]. Recently, a new RBF168 strain prototype of a lineage HIV-1 group P, closely related to SIVgor, was described in an old woman in Cameroon, indicating that gorillas, like chimpanzees, are probable sources of HIV-1 [17], [19].

Chimpanzees are classified into four subspecies on the basis of differences in mitochondrial DNA sequences, with a characteristic geographic distribution: *P. troglodytes verus* (*P.t.v*) in West Africa, *P. troglodytes ellioti* (*P.t.e*) [20] (formely termed *P. t vellerosus*) in Nigeria and northern Cameroon, *P. troglodytes troglodytes* (*P.t.t*) in southern Cameroon, Congo and Gabon, and *P. troglodytes schweinfurthii* (*P.t.s*) in the Democratic Republic of the Congo and the countries of East Africa [21]. The only two subspecies of chimpanzees found to be infected are *P.t.t* and *P.t.s* [4]. Despite extensive testing, naturally occurring lentiviruses have not been detected in West African chimpanzees (*P.t.v* or *P.t.e*), although one *P.t.e* (Cam 4) contracted SIV from a *P.t.t* in captivity [12], [22], [23]. The prevalence rate of SIVcpz varies considerably, ranging from 0 to 50% [22]–[24]. This finding, combined with the absence of SIVcpz in two of four subspecies, suggests that chimpanzee acquired the virus recently, before their differentiation into subspecies [25].

The origin of SIVcpz itself remains unclear [4], [26]. Phylogenetic analyses of the SIVcpz-US strain and seven other SIV lineages revealed that the SIVcpz genome has a recombinant origin. SIVcpz clustered closely with SIVrcm from red-capped mangabeys (*Cercocebus torquatus*) in the 5′ half of the genome, in Nef and in 3′LTR., and closely with SIV from several *Cercopithecus* species (*C.nictitans, C. cephus, C. mona*) in the *vpu, tat*, *rev*, and *env* genes [26], [27].

*P.t.t* in central Africa were thus recognized as a reservoir of SIVcpz*Ptt* viruses, which have been transmitted at least twice to humans, resulting in infections with HIV-1 groups M and N [5], [23], [25]. Interestingly, the HIV-1 group N is closely related to SIVcpzPtt-EK505 from Dja forest (south-central Cameroon) and HIV-1 group M to SIVcpzPtt-MB/LB from the south-eastern corner of Cameroon [22], [23]. The origin of emergences of HIV-1 group O and P remains unclear but was undoubtedly in the same area.

It was reported recently that chimpanzees can develop AIDS after natural infection with SIVcpz. Most of the documented cases have been found in *P.t.s* subspecies in Gombe National Park in Tanzania [28], resulting in a decline in the chimpanzee population in the area with the highest SIVcpz prevalence [24]. Recently, however, a case of natural SIVcpz infection in *P.t.t*, with clinical progression to AIDS-like disease, was reported [14].

Since 1994 a large survey of SIV prevalence in non-human primates in Gabon has been undertaken with synthetic peptide-based ELISA containing all known primate lentivirus lineages [29]. We recently found two new cases of SIVcpz in a wild-born orphan chimpanzee in Gabon and in a wild-born chimpanzee in Equatorial Guinea that had been seized as pets in Libreville, Gabon. We present here the characterization of a new SIVcpz strain, SIVcpz*Ptt*-Gab4, isolated from one of these chimpanzees. Additionally, to assess the degree of pathogenicity of natural SIVcpz infection in *P.t.t*, its clinical and immunological features were investigated.

## Results

### Serologic Survey and SIV Strains

We screened 104 wild-born chimpanzees in two central African countries, Gabon and Congo, for the presence of SIV antibodies (Table 1). Two sera, one from a chimpanzee (Gab3) in Haut-Ogooué Province and the second from a chimpanzee (Gab4) in Estuaire Province, reacted positively to HIV-1/SIVcpz-specific peptides in a specific peptide-based ELISA (see Methods). Gab3 was only 1 month old when it arrived, in February 2000, at the Primatology Centre (CIRMF), and it died 2 months later of unknown causes. Gab4 was seized with two other chimpanzees from their owner in Libreville in October 2006 for transfer to La Mouila Park (Bakoumba, southern Gabon). Because of its serological status, Gab4 was not introduced into the sanctuary and is now at the CIRMF Primatology Centre. Neither Gab3 nor Gab4 had hepatitis B or C or STLV infection.

**Table 1:** Numbers of samples collected from wild-born chimpanzees by geographic origin, sex and SIV status.

Country	Province	No. tested	Male	Female	SIV+
Gabon	Estuaire	4	2	2	1
	Haut-Ogooué	30	13	17	1*
	Moyen-Ogooué	6	4	2	0
	Nyanga	2	1	1	0
	Ogooué-Ivindo	5	0	5	0
	Ogooué-Lolo	5	3	2	0
	Woleu-Ntem	5	3	2	0
	Unknown	10			0
Congo	Konkouati	37	16	21	0
Total		104	42	52	2

* Juvenile that died at 3 months.

Gab4 showed the highest reactivity with SIVcpz*Ptt*-Gab1 and HIV-1 group N-specific peptides derived from and mapping to the env-V3 region and HIV-1 group N-, SIVcpz*Ptt*-Gab1- and SIVcpz*Ptt*-Gab2-specific peptides derived from and mapping to the env-gp41 peptides. The plasma of Gab3 at 1 month of age showed greater reactivity to HIV-1 group M V3 peptide than to HIV-1 group N and SIVcpz*Ptt*-Gab1 peptides and to the peptides mimicking gp41, the highest optical densities were found for HIV-1 group N and SIVcpz*Ptt*-Gab1. Plasma sampled at 2 months showed greater reactivity to SIVcpz*Ptt*-Gab1 V3 peptide and HIV-1 group N gp41 peptide. At 3 months, only weak reactivity was found to HIV-1 group N and SIVcpz*Ptt*-Gab1 gp41 peptides.

Analysis of the western blot profile revealed decreasing HIV-1 cross-reactivity with plasma from chimpanzee Gab3. High reactivity was detected on band C, corresponding to sampling at 1 month of age. Subsequent samples corresponding to bands D and E (Gab3 aged 2 and 3 months, respectively) clearly show progressive disappearance of antibodies. The virus could not be isolated or characterized, and western blot analyses suggested passive transmission of maternal SIV antibodies.

**Figure 1:**
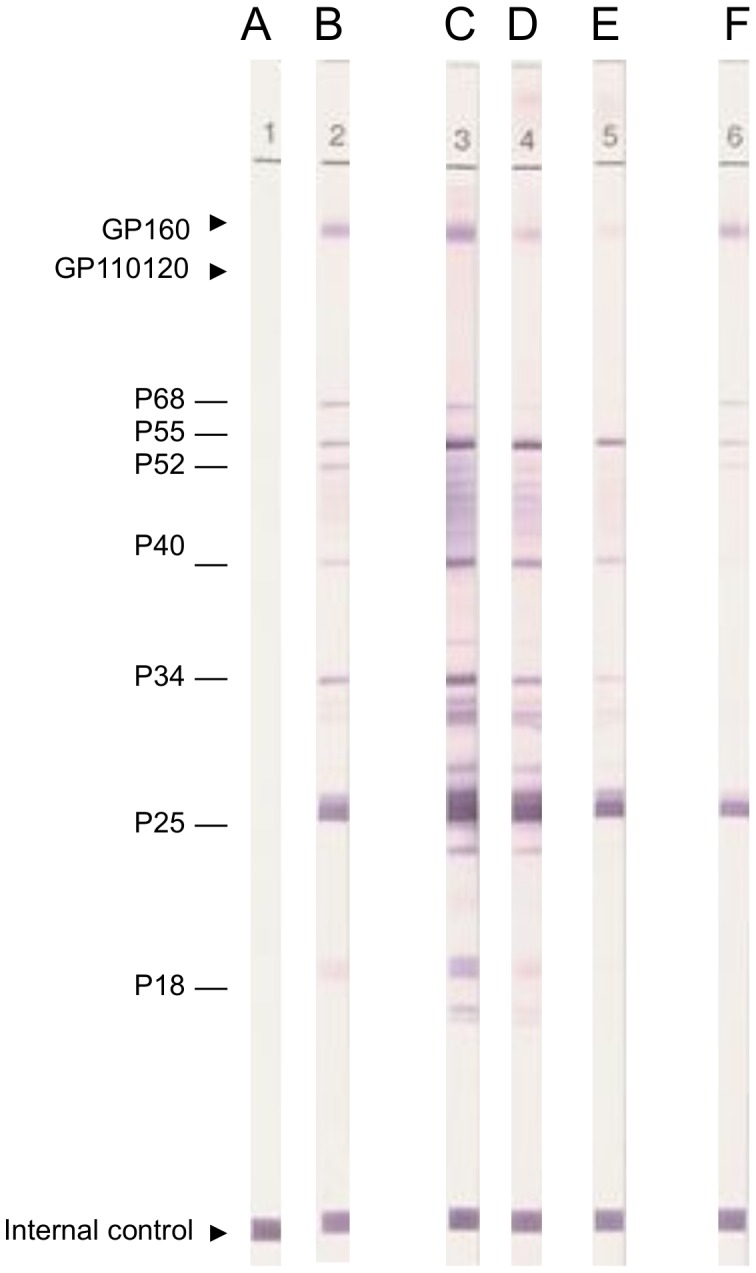
Western blot profiles of SIV-positive chimpanzees from Gabon. Plasma samples from chimpanzees Gab3 and Gab4 were tested for the presence of HIV-1 cross-reactive antibodies by western immunoblotting. Strips A and B are negative and positive controls, respectively. Progressive loss of HIV-cross-reactive antibodies in chimpanzee Gab3 is represented on strips C (at arrival, age 1 month), D (2 months), and E (death at 3 months). Strip F illustrates the HIV-1 cross-reactivity in plasma from chimpanzee Gab4.

Conversely, strong HIV-1 cross-reactivity was observed with plasma from chimpanzee Gab 4, particularly with the HIV-1 gp160 and p24 proteins, whereas the cross-reactivity with the remaining HIV-1 antigens was weaker (Figure 1). The SIVcpz*Ptt*-Gab4 virus was successfully isolated and the complete genome amplified and characterized.

### Identification of Chimpanzee Subspecies

To identify the origin and subspecies of our positive animals, the mtDNA fragment spanning the hypervariable D-loop region was characterized phylogenetically. The sequences obtained were compared with known sequences retrieved from the GenBank. The two new SIV-positive chimpanzees from Gabon (accession numbers, GQ915583 and GQ915584) were identified as members of a *P.t.t* subspecies interspersed among chimpanzee strains from Gabon, Cameroon and other central African countries (data not shown).

### Organization of the SIVcpz-Gab4 Genome

**Figure 2:**
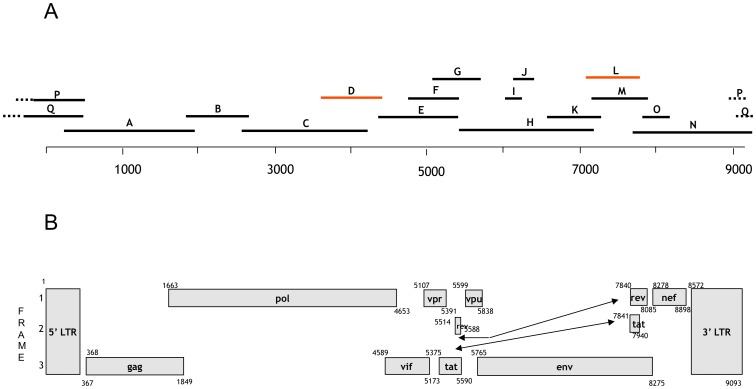
The Amplification strategy and the genome structure of the new SIVcpz identified strain. (A) Amplification strategy (primers listed in Table S1); (B) genome structure of SIVcpz-Gab4, depending on frame. Each gene is represented by a rectangular box with the name inside and its position within the genome. Arrows point to gene spread over two locations.

The complete SIVcpz*Ptt*-Gab4 sequence was obtained with a PCR-based ‘genome walking’ approach to generate 17 overlapping fragments. The genome was determined to be 9093 bp long. It is characterized by the presence of three retroviral structural genes (*gag*, *pol* and *env*) and the regulatory genes (*vif, rev, tat, vpr* and *nef*), including *vpu*. Complete open reading frames were found for all genes (Figure 2). The full genome sequences were analyzed gene by gene in MEGA and compared with SIVcpz sequences, in particular those of SIVcpz*Ptt*-Gab1 and Gab2.

The SIVcpz*Ptt*-Gab4 long terminal repeat comprised one binding site for NF-κB and two SP1 sites, the polyadenylation signal and TAATA box. No mutation was observed in gag p6 sequences in the PT/SAP domain of SIVcpz*Ptt*-Gab4 after comparison with all known SIVcpz/HIV-1 strains. The importance of the mutation at position 30 (Met-to-Arg) of the *gag* p17 protein during interspecies transmission of SIVcpz to humans has been demonstrated [30], [31]. The *gag* protein of SIVcpzGab4 has a methionine at position 30, and this is characteristic of all SIVcpz*Ptt*, indicating that they belong to the same phylogenetic group.

Moreover, instead of the YPSL motif found in SIVcpz*Ptt*-Gab2 strain, SIVcpz*Ptt*-Gab4 shared an LTSL motif, as did all the SIVcpzPtt/HIV-1 strains. There was also no mutation in the YMDD motif in SIVcpz*Ptt*-Gab4 *pol* sequences.

The *vpu* gene is made up of 80 amino acids and shows wide variation, like all SIVcpz strains. Neverteless, we found the DSGNES motif in the cytoplasmic domain of all SIVcpz*Ptt*. This highly conserved residue is involved in down-regulation of CD4 expression and in degradation of the BST-2 host restriction factor [32], [33], [34].

**Figure 3:**
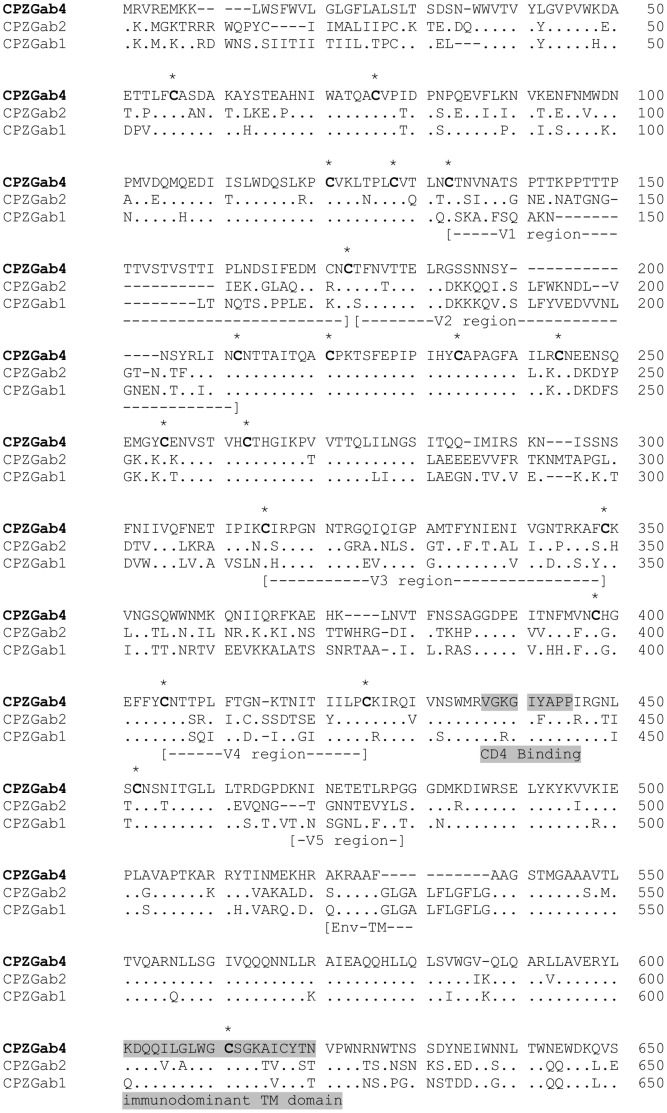
Predicted protein sequence of the *env* gene (gp120 and gp41) of SIVcpz-Gab4 in comparison with SIVcpz-Gab1 and SIVcpz-Gab2. Conserved cysteines are marked with asterisks, variable regions V1–V5 are indicated, and the CD4 binding site and immunodominant transmembrane domain are highlighted in grey.

In the *env* gene, the extracellular envelope domain (gp120) of SIVcpz*Ptt*-Gab4 contained V1, V2, V3, V4, and V5 regions, a CD4 binding site, and the cleavage site for the transmembrane glycoprotein in the gp41 domain. The V1 region was longer than V2 because of the deletion of 15 amino acids in V2. Moreover, detailed inspection of the amino acid sequence revealed the presence of 18 highly conserved cysteine residues, common to the three SIVcpz*Ptt*-Gab strains, which are involved in the formation of disulfide bonds and play an important role in the structure and function of gp120 (Figure 3).

The functional domains of the SIVcpz*Ptt*-Gab4 *rev* gene were conserved; one is rich in arginine (amino acids 35–50) and the other is rich in leucine (amino acids 73–83), as described for SIVcpz*Ptt*-Gab 1 and SIVcpz*Ptt*-Gab2 [9].

The *nef* gene contains 207 amino acids and groups of specific motifs with putative functional relevance. The consensus site of *N*-myristoyltransferase (MGXXXZ_6_) is present, as in all SIVcpz*Ptt* and HIV-1 strains [35]. Similarly, the site rich in proline PXXP representing the SH3 binding site is present [36], [37]. The EXXXLL_165_ motif, which is involved in down-regulation of CD4, is also conserved, as in other HIV-1 and SIVcpz strains [38].

### Phylogenetic Relation between the SIVcpz-Gab4 and Other Primate Lentiviruses

**Figure 4:**
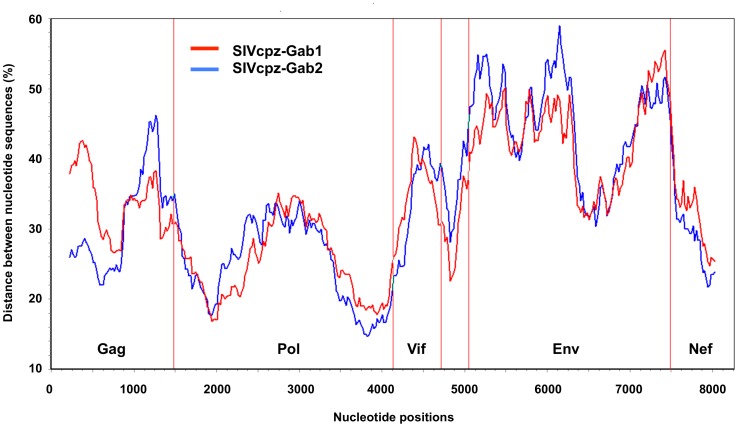
Diversity plot of SIVcpz-Gab4 with SIVcpz-Gab1 and SIVcpz-Gab2 sequences. Regions with uncertain aligment or sites with a gap in any sequence were excluded (8261 nucleotides after de-gapping). The nucleotide sequence difference is plotted for windows of 450 nucleotides and a 20-nucleotide step increment.

We examined the phylogenetic relation between the SIVcpz*Ptt*-Gab4 strain and other primate lentiviruses and then the relation with SIVcpz*Ptt*-Gab1 and SIVcpz*Ptt*-Gab2 strains, by diversity plot analyses of concatenated nucleotide sequences. Pairwise sequence distances were plotted for windows of 450 nucleotides, which were moved in steps of 20 nucleotides along the alignment. SIVcpz*Ptt*-Gab4 clustered closely with SIVrcm (red capped mangabey, *Cercocebus torquatus torquatus*) in *pol* and closely in *env* with SIVgsn (greater spot-nosed monkey, *Cercopithecus nictitans*), as did other SIVcpz strains (data not shown). As seen in Figure 4, the SIVcpz*Ptt*-Gab strains showed the greatest homology of sequences across the genome, but the homology varied within genes. The results show crossing of diversity plots, indicating the presence of recombination events characteristic of mosaic genomes.

### Phylogenetic Relations of SIVcpzPtt-Gab4

**Figure 5:**
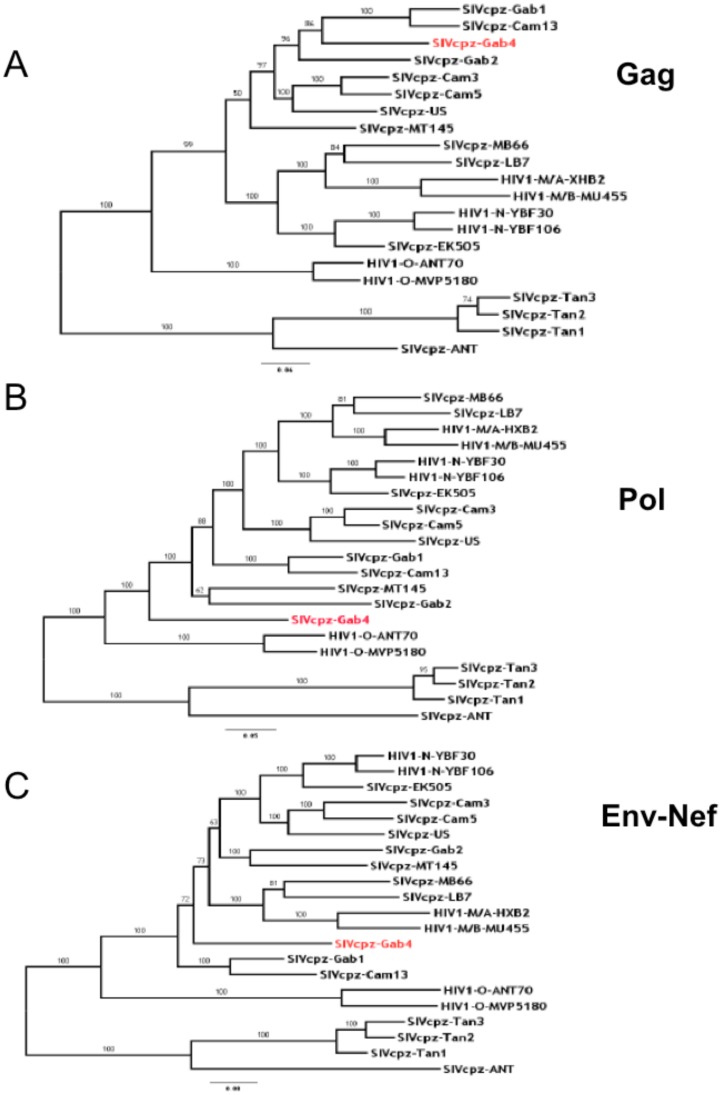
The phylogenetic relations between the new SIVcpz*Ptt*-Gab4 strain and other SIVcpz strains. A: SIVcpz-Gab4 *gag*. Bayesian method implemented in MrBayes, run for 500 000 generations with a 2.5% burn-in. Mixed model in MrBayes indicated rtREV model of amino acid change with gamma distribution rates across sites. Parameters examined with Tracer program with an effective sample size of 1308. B: SIVcpz-Gab4 *pol-vif-AA*. Bayesian method implemented in MrBayes, run for 500 000 generations with a 2.5% burn-in. Mixed model in MrBayes indicated Jones model of amino acid change with gamma distribution rates across sites. Parameters examined with Tracer program with an effective sample size of 1118. C: SIVcpz-Gab4 *env-nef*. Bayesian method implemented in MrBayes, run for 500 000 generations with a 2.5% burn-in. Mixed model in MrBayes indicated rtREV model of amino acid change with gamma distribution rates across sites. Parameters examined with Tracer program with an effective sample size of 936.

To estimate the phylogenetic relations between the new SIVcpz*Ptt*-Gab4 strain and other SIVcpz strains, we constructed and analyzed the maximum likelihood phylogenetic trees from the *gag*, *pol–vif* and *env–nef* amino acid sequences (Figure 5). The position of the SIVcpz*Ptt*-Gab4 strain varied: in the *gag* tree (Figure 5A), it belonged to the SIVcpz*Ptt*-Gab1, SIVcpz*Ptt*-Cam13 and SIVcpz*Ptt*-Gab2 group, supported by a strong (96%) bootstrap value; in the *pol–vif* tree (Figure 5B), it was an outlier to the entire clade, which was composed of other SIVcpz*Ptt* and HIV-1 groups M and N (bootstrap value, 100); and in the *env–nef* tree (Figure 5C), it was within the group of strains that includes SIVcpz/HIV-1 group M and HIV-1 group N, but with no strong relation to any of the strains present (bootstrap value, 72).

Comparison of the predicted protein sequences encoded by the *gag, pol–vif* and *env–nef* genes revealed that SIVcpz*Ptt*-Gab4 is more closely related to SIVcpz*Ptt*-Gab1 and SIVcpz*Ptt*-Gab2 in the genes analyzed. Thus, as shown in Table 2, the percentage protein sequence identities were: in *gag*, 82.4% with SIVcpz*Ptt*-Gab2 and 81.3% with SIVcpz*Ptt*-Gab1; in *pol–vif*, 78.3% with SIVcpz*Ptt*-Gab2; and in *env–nef*, 67.0% protein sequence identity with SIVcpz*Ptt*-Gab2.

**Table 2:** Protein sequence identities among SIVcpz/HIV-1 viruses.

Strain compared withSIVcpz-Gab4	Amino acid identity (%)		
	Gag	Pol-vif	Env-nef
SIVcpz-Gab2	**82.4**	72.0	62.5
SIVcpz-Gab1	81.3	**78.3**	**67.0**
SIVcpz-Cam	80.3	76.4	64.4
HIV-1-N	75.6	75.7	64.1
HIV-1-M	73.3	76.7	59.6
HIV-1-O	71.5	73.7	41.7
SIVcpz-Tan	61.7	64.2	36.5

### Virus Isolation, Monitoring of Viral Replication, and Quantification of SIVcpz RNA

No SIV was detected after culture of PBMCs from chimpanzee Gab3. Real-time PCR for detection of the plasma viral load and the proviral load were negative. SIV was, however, isolated after T-cell depletion from PBMCs of chimpanzee Gab4, on day 11 of in vitro culture, confirming SIV infection in this chimpanzee. p24 antigen and RT activity were detected in supernantants collected between days 3 and 17, with a peak at day 11. p24 antigen was also detected in the plasma of this infected animal. The plasma viral load of this chimpanzee on arrival at the CIRMF was 9.2×10^3 ^RNA copies/ml, and no significant difference in viral load was found during 3 years of follow-up (Table 3).

**Table 3:** Hematologic and immunologic parameters of chimpanzee Gab4 and of 16 uninfected chimpanzees.

Variable	Time of follow-up			
	At arrival	6 months	2 years	Uninfected chimpanzees (mean of 16±SD)
Age (years)	6	6	8	8.8±2.9
Sex	M			11M, 5F
Plasma viral load (RNA copies/ml)	4.5×10^3^	2.5×10^3^	1.9×10^3^	
White blood cells	8.2	4.1	5.8*	9.3±2.8
Red blood cells	6.0	4.7	5.8	5.3±0.9
Platelets	483	363	345	351±144
% CD4^+^ T cells	22.7	25.1	25.7*	38.8±8.8
CD4^+^ T cells (number/mm^3^)	149	268	283*	534±196
% CD8^+^ T cells	28.2	34.4	25.8	26.2±8.8
CD8^+^ T cells (number/mm^3^)	185	367	284.3	363±158
CD4^+^ T cells/CD8^+^ T cells (ratio)	0.8	0.7	1.0	1.7±0.8
%HLA-DR in CD4^+^ T cells	16.5	3.0	1.1	1.9±1.3
%HLA-DR in CD8^+^ T cells	28.8	12.3	4.8	6.1±0.3
%Ki67 in CD4^+^ T cells	ND	ND	4.0	2.4±1.0
%Ki67 in CD8^+^ T cells	ND	ND	4.3	2.1±1.3
%CD25 in CD4^+^ T cells	50.3	55.2	44.0	36.9±8.8
%CD25 in CD8^+^ T cells	3.4	2.7	2.9	4.4±2.6
% CD28^+^CD95^−^ in CD4^+^ T cells (naives)	55.0	52.2	69.0	53.4±17.8
% CD28^+^CD95^+^ in CD4^+^ T cells (CM)	44.5	46.5	29.0	41.8±13.9
% CD28^−/+^CD95^-^ in CD4^+^ T cells (EM)	0.5	1.3	2.1	4.8±5.9
% CD28^+^CD95^-^ in CD8^+^ T cells (naives)	26.0	19.7	21.9	31.3±16.1
% CD28^+^CD95^+^ in CD8^+^ T cells (CM)	23.5	16.4	25.5	17.1±6.2
% CD28^−/+^CD95^-^ in CD8^+^ T cells (EM)	50.5	64.0	52.7	53.4±15.2

CM, central memory; EM, effector memory.

* p<0.05 determined with Mann-Witney *U* test.

### Follow up of Hematologic and Immunologic Parameters in SIV-infected Chimpanzee Gab4

We evaluated the hematologic and immunologic effects of SIV infection in chimpanzee Gab4 and compared them with those of 16 uninfected chimpanzees housed at the CIRMF (Table 3). Of the basic hematologic markers, only the number of white blood cells was significantly lower than that in uninfected animals (*p*<0.05). Analysis of T-cell subsets showed a significantly low percentage and absolute number of CD4+ T cells (*p*<0.05). No significant difference was found in CD8+ T cells, and no difference was found in the distribution of naive or memory cells or in the proliferation and activation of CD4+ and CD8+ T cells.

## Discussion

During routine HIV/SIV screening of non-human primates, we identified two chimpanzees harboring anti-SIVcpz antibodies. With the previously described and characterized SIVcpz-Gab strains [6], [9], SIVcpz*Ptt*-Gab4 represents the third SIVcpz strain identified and characterized in wild-born captive chimpanzees in Gabon, central Africa. The absence of virus in the newborn seropositive chimpanzee Gab3 and the progressive disappearance of cross-reactive HIV-1 antibodies strongly suggests passive transplacental transfer of antibodies from infected mothers, as observed in infant chimpanzees [1]. Interestingly, Gab3 and Gab4 showed different serological patterns. As we did not have the original strain from the mother of Gab3, we can only hypothesize that Gab3 and Gab4, which were from different geographic areas, are sufficiently different that they induce distinct serological profiles. In a large study on the natural prevalence of SIVcpz, antibodies detected in urine and feces also showed different profiles, linked to viral diversity and, to a lesser extent, to the sites at which the samples were collected [23].

Analysis of mtDNA (D-loop) sequences showed that our SIV-positive chimpanzee Gab4 belongs to the *P.t.t* subspecies. Once we had sequenced the entire SIVcpz*Ptt*-Gab4 genome, we found that the new strain has the same genomic organization found in all HIV/SIVcpz [39].

We evaluated the sequence homologies of SIVcpz*Ptt*-Gab4 with two previously characterized Gabonese SIVcpz strains by phylogenetic analyses of evolutionary trees constructed for the three main genes. As reported for SIVcpz*Ptt*-Gab1 and SIVcpz*Ptt*-Gab2 [7], [9], SIVcpz*Ptt*-Gab4 is phylogeneticaly related to the SIVcpz*Ptt*/HIV-1 lineage rather than to SIVcpz*Pts*, but its position varies. The strongest amino acid identities were observed with SIVcpz*Ptt*-Gab2 and SIVcpz*Ptt*-Gab1. Interestingly, despite the relatively close similarity, clustering of these SIVcpz*Ptt*-Gab strains was supported by a strong 96% bootstrap value only for the *gag* tree.

Global analysis of the phylogenetic results showed high genetic diversity among the Gabonese SIVcpz*Ptt* strains. As reported previously [13], SIVcpz*Ptt*-Cam13 clustered more closely with SIVcpz*Ptt*-Gab1 than with SIVcpz*Ptt*-Gab2; in the present study, it clustered with the newly characterized SIVcpz*Ptt*-Gab4 strain. This might be due partly to the origin of the two chimpanzees: Gab1 was from northern Gabon and Cam13 from a neighboring province in Cameroon, with no significant biogeographic barrier between the two. Conversely, the second SIV-positive chimpanzee, Gab2, was from eastern Gabon, isolated from the two others by the Ogoouée and Ivindo rivers. Equatorial Guinea, the place of origin of Gab4, represents an intermediary geographic locality, with no hindrance to chimpanzee movement.

In contrast, the SIVcpz strains from Cameroon fell into the specific *SIVcpzPtt-*Cam cluster in the *gag, pol*, and *env* trees, as did the SIVcpz*Ptt*-US strain from an unknown African country [5], [13], [22], [23]. In this particular cluster, however, SIVcpz*Ptt*-Cam strains differ according to their geographical origin and some appear to be particularly closer to HIV-1 group M or N [22], [23]. These phylogenetic data strongly suggest that HIV-1 group M emerged from the south-eastern corner of Cameroon while HIV-1 group N appeared in south-central Cameroon [25].

The importance of host restriction factors in the natural history of SIV and the emergence of the HIV pandemic was demonstrated recently. These factors include APOBEC3G [40], TRIM5α [41] and tetherin (also known as BST-2 or CD137), which inhibits the budding and release of viruses in cells [42]. The authors showed that several SIVs use *nef* to block the expression of tetherin on the cell surface by targeting its cytoplasmic domain [43], [44]; however, HIV-1 and SIVmon, SIVgsn and SIVden use *vpu* to degrade tetherin [42], [45], [46]. This difference in response is the result of specific host selection pressure after interspecies transmission [25], [45]. Sharp et al. therefore proposed that, in order to replicate in humans, SIVcpz has to find an alternative, means to counteract the activity of tetherin, such as use of *vpu*. The *nef* gene of SIVcpz*Ptt*-Gab4 might have the same tetherin inhibition functions as other SIVcpz*Ptt* in its natural host but also the capacity to downregulate the expression of CD4 and MHC-1 at the surface of human cells, without undergoing adaptation [35]. Despite these theoretical transmission capabilities, to date, none of the Gabonese SIVcpz*Ptt* strains appears to be the original reservoir of the HIV-1 group.

Within a project for the epidemiological surveillance of primates in central Africa (Grant ROI AI44596) that began in 2000, we have systematically screened all samples taken from monkeys kept as pets representing 13 species of primate [47] including 104 chimpanzees. This type of sampling does not reflect the prevalence of SIVcpz*Ptt* in Gabon, for which noninvasive studies involving the collection of faeces in Cameroon and Tanzania provide more convincing evidence [22]–[24], [48]. Nevertheless, our study confirms the existence of SIV-infected chimpanzees circulating in northern Gabon and Equatorial Guinea (SIVcpz*Ptt*-Gab1, SIVcpz*Ptt*-Gab4), eastern Gabon (SIVcpz*Ptt*-Gab2), and southern Gabon (*P.t.t* Gab3). These geographic indications will be important for planning future non-invasive studies.

Few studies have been conducted of the pathogenesis of SIV in chimpanzees, for two main reasons. First, many SIV strains are known only from the available sequences and have not been isolated. Secondly, chimpanzees are endangered non-human primates and their use in experimental studies is prohibited. Only two *P.t.s* infected with SIVcpz*Pts*-Ant have been monitored for several years [49]. We were able to study the chimpanzee Gab4 at the CIRMF where it is housed and could thus evaluate its immunological and virological parameters. No sign of disease associated with SIV has been identified during the 2 years of the survey. The level of viral replication in plasma was low, at about 4 log_10_; however, the only other known viral loads are those of two chimpanzees, Cam 155 and Noah, which are 3.4–5.8 log_10_ [14], [50]. Furthermore, the assumption that African primates infected with SIV have high viral loads is now in doubt, as several studies have shown wide variation among individuals of the same species, especially sooty mangabeys and African green monkeys [51], [52]. It is therefore difficult to define a general viral load for all chimpanzees infected with SIVcpz.

No significant variation was found in the viral load of Gab4 during the study. The CD4/CD8 ratio also showed no significant variation, indicating that the moderate viral replication recorded in Gab4 did not impair its immune system. Only slight leukopenia and a lower level of CD4+ T cells were observed when compared with uninfected chimpanzees. Depletion of CD4+ T cells is not the only factor involved in disease progression, however, and chronic immune activation is a very strong predictor of pathogenic SIV infection [53], [54]. SIVcpz*Ptt*-Gab4 infection was associated with neither T-cells proliferation nor T-cells activation. This correlates with the stable health of this animal and the absence of signs of immunodeficiency.

Gab4 is the oldest *P.t.t* infected with SIVcpz known in Gabon. All the animals were at least 2 years old when they were found to be seropositive, and Gab4 was 6 years old at that time. As it was not old enough to be sexually active, it was probably infected by maternal-fetal transmission, like the other chimpanzees [28]. By the end of our study (2008) this animal had been infected for 8 years. Of the 10 chimpanzees studied to date, five died before the age of 6 years (Cam13, Cam5, Cam3, Gab1, Gab2) and one after 20 years (Marilyn). Two are still alive and in good health at over 20 years of age (Cam4 and Noah). The other chimpanzee (Cam155) is still alive at 7.7 years but is in a pre-AIDS stage, corresponding to WHO stage III in humans [14].

Recent immunopathological studies in communities of *P.t.s* in Tanzania showed that SIVcpz*Pts* is also associated with progressive CD4+ T-cell loss, lymphatic tissue destruction and premature death. SIVcpz*Pts*, correlated with high prevalence, has a substantial negative influence on the health, reproduction and lifespan of chimpanzees in the wild [28], [55]. All these data suggest that SIVcpz is generally nonpathogenic but can induce pathogenic effects under certain circumstances. This is consistent with the idea of an increased susceptibility because of the recent introduction of SIVcpz into chimpanzees [56].

We have reported here the identification and characterization of a new SIVcpz*Ptt* strain, SIVcpz*Ptt*-Gab4, which is thus the third SIVcpz*Ptt*-Gab strain from a chimpanzee captured in the wild in Equatorial Guinea and then living in captivity in Gabon. It is interesting to note the strong genetic diversity that characterizes the three Gabonese SIVcpz strains. Up to now, no reliable clinical symptoms of SIV infection have been detected in the SIV-positive chimpanzee. Further field studies with non-invasive detection methods are needed to determine the prevalence, geographic distribution, species association and natural history of SIVcpz strains throughout the chimpanzee habitat in Gabon.

## Materials and Methods

### Chimpanzee Collection at CIRMF

Within a project for the epidemiological surveillance of primates in central Africa (Grant ROI AI44596) that began in 2000, we have systematically screened all samples taken from monkeys kept as pets comprising 13 species of primates (Mouinga-Ondeme et al., 2012), including 104 chimpanzees. As shown in Table 1, the serologic survey involved 37 wild-born orphaned chimpanzees from the Habitat Ecologique et Liberté des Primates (HELP) Sanctuary in Conkouati-Douli National Park, Congo, from which plasma samples were collected during routine veterinary screening in 1992 and 1996 and 67 wild-born chimpanzees sampled in the wild throughout Gabon. All the animals were negative, except for Gab3 and Gab4, which were seropositive for SIV.

### Ethics Statement

The animals were handled in accordance with standard national operating procedures in the CIRMF as well as in accordance with the United States National Institutes of Health guidelines for the Care and Use of Laboratory Animals. The animal protocols and procedures were approved by the Gabonese ethics committee for animal experimentation at the CIRMF, and registered under No. CE08–010.

All work with animals was conducted according to the relevant national and international guidelines and in accordance with the recommendations of working group report chaired by Sir David Weatheall in December 2006 and Guide for the Care and Use of Laboratory Animals of the National Institutes of Health. CIRMF collaborates with great apes sanctuaries to perform routine serological surveys of adopted orphans. In order to integrate animals safely, new orphans designated for a sanctuary or a releasing project are admitted to the CIRMF Primate Centre Quarantine Facilities, which is an unique national health primate reference centre approved by the Gabonese Wild Fauna and Agricultural Services and, the Ministry of Research. Veterinarians at Primate Centre have examined animals and researchers in the Retrovirus Department have performed serological analyses for 20 years.

The housing conditions are in strict accordance with European Union guidelines for animal care (European Union Directive 86/609/EEC). The Primate Centre has spacious rooms equiped with branches, hammock, platforms and ball toys, which are changed regularly. Animal welfare ensure to prevent suffering in all work involving non-human primates: e.g. they are fed twice a day with various Gabonese fruits and with a “home-made” protein complement cake. Food enrichment and training (positive reinforcement) practised dayly to obtain cooperation for routine veterinarian examinations. Highly skilled staff spends 2 h a day with the non-human primates.

Each primate housed in the CIRMF Primate Centre has an annual health check under anesthesia (ketamine at 10 mg/kg body weight). The blood samples, taken for this study, were collected under strict health controls by Primate Centre veterinarians.

### Specimen Collection

Blood samples from SIV-positive chimpanzees were collected in EDTA K2 tubes under ketamine-HCl (10 mg/kg bw) and used for flow cytometry. Peripheral blood mononuclear cells (PBMCs) were isolated by Ficoll-Hypaque gradient centrifugation (Sigma-Aldrich), and plasma was centrifuged at 3000×*g* for 10 min, dispensed into 1-ml aliquots and frozen at –80°C.

### Detection and Confirmation of SIV Antibodies

Plasma samples from all animals were first screened with the Determine HIV-1/2 rapid test (Alere Inc, San Diego, CA, USA). Positive samples were tested with the peptide-based primate lentivirus identification assay [29]. This indirect ELISA method is based on use of synthetic peptide antigens that map to the immunodominant gp41 region and the V3 region of HIV/SIVcpz reference strains: HIV-1 group M subtype A (consensus), HIV-1 group O (Ant-70), HIV-1 group N (YBF30), SIVcpz*Ptt*-Gab1 (Gab1) and SIVcpz*Ptt*-Gab2 (Gab2). All positive and equivocal samples were subjected to western blotting confirmation (New Lav Blot 1, Biorad, Marnes la Coquette, France).

### Amplification of SIVcpz Viral RNA by RT-PCR

The complete SIVcpz*Ptt*-Gab4 genome was amplified from RNA extracted from the plasma of the infected chimpanzee. PCR amplification was performed on a thermal cycler Perkin Elmer 9700. We first used degenerated primers to amplify a 330-bp fragment of *pol* (PoliS4 and PolOR for the first round and Hpol4235-Hpol4538 for the second round) and a 550-bp fragment of *env* (gp40F1 and gp41R1 for the first round and gp46F2 and gp47R2 for the second round) with RT-PCR, as described previously [57]–[59]. The PCR products were directly sequenced. We then amplified the full-length provirus by the long-PCR procedure (GeneAmp XL kit Perkin Elmer, Norwalk, Connecticut, USA) with two sets of primers, LPBS15’–Hpol4538 and Lsigi3’–Hpol4235, under the cycle conditions described previously [11] [12], [60]. Briefly, amplification comprised a hot start (2 min at 94°C), 16 cycles of denaturation (94°C, 30 s), hybridization (55°C, 1 min) and extension (68°C, 10 min), followed by 24 cycles of denaturation (94°C, 30 s), hybridization (55°C, 1 min), and extension (68°C, 10 min with increments of 30 s at each cycle). To obtain the full-length viral sequences, a nested PCR was performed with 5 µl of the PCR product and combinations of different sets of universal and/or virus-specific primers (Table S1).

The PCR products were purified and directly sequenced (GATC Biotech, Konstanz, Germany). The full-length SIVcpz*Ptt*-Gab4 sequence was deposited in GenBank (accession number, GQ217539).

### Species and Sub-species Determination (mtDNA-D loop)

To confirm the subspecies origin of the SIV-positive chimpanzees, a 341-bp region of the mtDNA genome (D-loop) was amplified, as previously described [61], with the primers L15997 5′-CACCATTAGCACCCAAAGCT-3′ and H16498 5′-CCTGAAGTAGGAACCAG.

ATG-3′. The resulting PCR products were directly sequenced (Macrogen Inc., Kumchun-ku, Republic of Korea).

These new mtDNA sequences were compared with those found in the GenBank database originating from 25 chimpanzees from Gabon and 26 from Cameroon, the Congo, the Central African Republic and the Democratic People’s Republic of the Congo, all representatives of *P. troglodytes troglodytes* subspecies. Of the 25 mtDNA sequences from Gabon, 16 originated from pets or wild-born, orphaned chimpanzees sampled within the country, and nine were from wild chimpanzees in the Lope Reserve. The samples were obtained by noninvasive methods [62]. mtDNA sequences characterizing the remaining *P. troglodytes* subspecies (*P. troglodytes schweinfurthii*, *verus*, and *ellioti*) and *P. paniscus* were also included.

Sequences were aligned with CLUSTAL W (1.7); all ambiguous sites with a gap in any sequence were excluded. Phylogenetic trees were constructed by the Bayesian method with MrBayes version 3.1 software (2005) [63] and the GTR model for gamma distributed rates at sites and one million generations with a burn in of 2.5%. Bayesian parameters were examined with the Tracer program (http:/evolve.zoo.ox.ac.uk/software.html/id=tracer); all the estimated sample sizes were greater than 220. The mtDNA chimpanzee sequences were deposited in GenBank (accession numbers, GQ915583and GQ915584).

### Phylogenetic Analyses

Pairwise alignments were performed for the nucleotides and deduced amino acids of separated SIVcpz*Ptt*-Gab4 genes (*gag, pol–vif* and *env–nef*) with CLUSTAL W (1.7), which constructs neighbor-joining trees in a Kimura two-parameter model (transition/transversion ratio = 2) [64]. The SIVcpz*Ptt*-Gab4 sequences were aligned with the corresponding sequences of representative SIVcpz and HIV-1 strains. The GenBank accession numbers were: HIV-1 MU455, M62320; HIV-1M HXB2, K03455; HIV-1N YBF30, AJ006022; HIV-1N YBF106, AJ271370; HIV-1O ANT70, L20587); HIV-1O MVP5180, L20571); SIVcpz-Gab1, X52154; SIVcpz-Gab2, AF382828; SIVcpz-Cam3, AF115393; SIVcpz-Cam5, AJ271369; SIVcpz-Cam13, AY169968; SIVcpz-US, AF103818; SIVcpz-TAN1, AF447763; SIVcpz-TAN2, DQ374657; SIVcpz-TAN3, DQ374658; SIVcpzANT, U42720; SIVcpz-MT145, DQ373066; SIVcpz-MB66, DQ373063; SIVcpz-LB7, DQ373064 and SIVcpz-EK505, DQ373065. The concatenated amino acid alignments were used for the phylogenetic analysis after exclusion of all sites that could not be aligned unambiguously or sites with a gap in any sequence and after removing the *gag/pol* and *pol/vif* overlaps from the C-terminus of the deduced *gag* and *pol* protein sequences. Trees were inferred by the Bayesian method implemented in MrBayes version 3.1 software (2005) [63] with the Jones, Taylor and Thornton model [65] and the Rtrev model [66] of evolution and gamma distributed rates at sites, with one million generations and burn-in of 2.5%. Bayesian parameters were examined with the Tracer program (http:/evolve.zoo.ox.ac.uk/software.html/id=tracer), and all estimated sample sizes were greater than 545.

### Virus Isolation and Viral Replication Monitoring

A portion of the PBMC was used for virus isolation. CD8 depletion was performed on 10 million lymphocytes with magnetic beads coupled to CD8 antibody, as recommended by the manufacturer (Dynabeads, Invitrogen Dynal, AS, Oslo, Norway). After washing, the enriched CD4+ cells were suspended in RPMI 1640 growth medium (Cambrex Bioscience, Walkersville, Maryland, USA) supplemented with 20% heat-inactivated foetal bovine serum (Gibco BRL, Eragny, France), 1% penicillin–streptomycin mixture (Gibco BRL, Eragny, France), 1% L-glutamine 200 mmol/l (Gibco BRL, Eragny, France), and 20 U/ml human recombinant interleukin-2 (Roche Diagnostics, Manheim, Germany). The lymphocytes were stimulated with 3 µg/ml of the mitogen concanavalin-A (Sigma-Aldrich, Saint Quentin Fallavier, France) and incubated at 37°C in 5% CO_2_. To maintain the cells, 50% of the medium was changed twice a week.

The second portion of PBMC was aliquoted in 10% DMSO (Sigma-Aldrich) in foetal bovine serum (Gibco-BRL) and frozen at –80°C.

Viral replication was monitored with a reverse transcriptase (RT) assay (Lenti-RT Kit, Cavidi Tech AB, Uppsala, Sweden) and by measuring p24 antigen (Genetic Systems HIV-1 Ag EIA, Biorad, Marnes-la-Coquette, France).

### RNA and DNA Extraction

RNA was extracted from 150 µl plasma with a QiaAmp Viral RNA Mini kit (Qiagen) and eluted in 60 µl TE buffer, as recommended by the manufacturer.

DNA was extracted from PBMCs with a QiaAmp DNA Mini kit (Qiagen) and eluted in 200 µl AE buffer.

### Quantification of SIVcpz RNA

Quantification by real-time RT-PCR was performed with 5 µl extracted RNA with a QuantiTect SYBR Green RT-PCR kit (Qiagen) in capillary tubes, by the LightCycler System (Roche Diagnostics).

Quantification was based on amplification of a 119-bp fragment located in the long-terminal-repeat region of the HIV-1 major group. We used the forward and reverse primers (AF) 5′-GCCTCAATAAAGCTTGCCTTGA-3′ (66–87) and (BR) 5′-GGCGCCACTGCTAGAGAT.

TTT-3′ (163–184) [67] and inactivated HIV virus (5×10^6 ^RNA copies per ml) as the standard (Biocentric, Bandol, France). The primers were used at a final concentration of 1 µmol/l, and the final MgCl_2_ concentration was 2.5 mmol/l. The amplification protocol for SIVcpz quantification consisted of reverse transcription (30 min at 50°C), followed by denaturation and activation of HotStart *Taq* DNA polymerase (15 min at 95°C) and cDNA amplification (45 cycles of denaturation for 15 s at 95°C, annealing for 15 s at 55°C, and elongation for 22 s at 72°C). The RNA copy number was determined by comparison with an external standard curve and was expressed as RNA copies per ml plasma. The detection limit of the SIVcpz quantification assay was 100 RNA copies per ml plasma.

### Flow Cytometric Analysis of Cell-surface and Intracellular Marker Expression

In addition to Gab4, we selected 16 uninfected chimpanzees aged 5–10 years and analyzed whole blood samples by four-color flow cytometry with a standard procedure and a panel of monoclonal antibodies: anti-CD4-fluorescein isothiocyanate (FITC) (clone MT4-77), anti-CD4-phycoerythrin (PE) (clone L200), anti-CD3-allophycocyanin (clone SP34-2), anti-CD8-peridine chlorophyll protein (clone SK1), anti-HLA DR-PE (clone G46-6), anti-CD25-PE (clone 2A3), anti-Ki67-FITC (clone B56), anti-CD28-PE (clone L293), and anti-CD95-FITC (clone DX2), all obtained from BD Bioscience (Le Pont de Claix, France). At least 10 000 events were acquired in the lymphocyte square on a FACScalibur flow cytometer driven by the CellQuest software package (Becton Dickinson, Heidelberg, Germany). Data were analyzed with FlowJo software v7.2 (Tree Star, Inc., Ashland, Oregon, USA).

### Statistical Analysis

The Mann-Whitney U test was used to compare the results of flow cytometry. Significance was assumed at *p*<0.05. All analyses were performed with Statistica software v7.1. (StatSoft France, www.statsoft.fr).

## Supporting Information

Table S1 Oligonucleotide primers used to amplify SIVcpz-Gab4 genome.
(DOC)
