# Supplementary material for: New Strain of Simian Immunodeficiency Virus Identified in Wild-Born Chimpanzees from Central Africa
Source: PLoS One. 2012 Sep 12;7(9):e44298. doi: 10.1371/journal.pone.0044298 (PMC3440395; doi:10.1371/journal.pone.0044298)
Supplement: Table S1 — Oligonucleotide primers used to amplify SIVcpz-Gab4 genome. (DOC) [file pone.0044298.s001.doc]

Table S1: Oligonucleotide primers used to amplify SIVcpz-Gab4 genome.

| Fragment  (a) | Forward primer  (name) | Sequence (b)  (5’ – 3’) | Position | Reverse primer  (name) | Sequence (b)  (5’ – 3’) | Position | Size  (c) |
| --- | --- | --- | --- | --- | --- | --- | --- |
|  |  |  |  |  |  |  |  |
| A | LTR5’V | AAAATCTCTAGCAGTGGCGCCCGAACAGG | 163-192 | DR100-inR | TGGARACCAAAAATGATAGG | 1945-1964 | 1782 |
| B | DR1 | TRCAYACAGGRGCWGATGA | 1892-1910 | DR2 | CARTAYATGGATGAYHTIT | 2662-2680 | 770 |
| C | DR100-outF | AATGTTYTRCCACAAGGATGG | 2557-2577 | Pol100-outR | ACAATTTTAAAAGAAAAGGGG | 4325-4345 | 1768 |
| D* | PoliS4 | CCAGCNCACAAAGGNATAGGAGG | 3727-3749 | PolOR | GAAAGGDGAAGGNGCRGTVGT | 4506-4523 | 779 |
| E | Pol100-inF | TGTGGAAAGGTGARGGGGCAG | 4502-4522 | REMOAS | TCCTATGGCAGGAAGAAGCG | 5510-5529 | 1008 |
| F | Tat100-inF | GGTAAGTATAGARTGGAGAC | 4840-4859 | REMOAS | TCCTATGGCAGGAAGAAGCG | 5510-5529 | 670 |
| G | Rev100-F1 | TATGGAGATACTTGGGAAGGAGT | 5254-5276 | Rev100-R1 | GAAAGAGCAGAAGATAGTGGAAAT | 5743-5766 | 489 |
| H | REVMS | TCCTATGGCAGGAAGAAGCGG | 5510-5530 | Gp100-2R | AGGAAGCACTATGGGCGCAG | 7258-7277 | 1748 |
| I | CpzEnvF1 | GACCAAATGCAGGAGGACATTAT | 6056-6078 | CpzEnvR1 | TGCATTGCACACATGGAATTAAAC | 6483-6506 | 427 |
| J | CpzEnvF2 | GATCAGAGCCTCAAACCCTGTGTA | 6089-6112 | CpzEnvR2 | ACACATGGAATTAAACCAGT | 6491-6510 | 402 |
| K | CpzEnvF3 | AGGCCAGGAAACAATACAAGAGGA | 6638-6661 | CpzEnvR3 | CGGCAGTTACGCTGACGGTACA | 7278-7299 | 640 |
| L* | gp40F1 | TCTTAGGAGCAGCAGGAAGCACTATGGG | 7245-7272 | gp41R1 | TTAGGCAGGGATACTCACCTCTCTCGTT | 7809-7836 | 564 |
| M | CpzEnvF4 | CGGCAGTTACGCTGACGGTACA | 7278-7299 | CpzEnvR4 | CGTTGCAGACCCTTATCCCAG | 7833-7853 | 555 |
| N | Gp100-inF | ATGGTTGTGGTATATTAAAAT | 7717-7737 | Lsigi1-3’ | CTGCTTAAGCCTCAATAAAGCTTGCCTTGA | 58-87 | 1434 |
| O | CpzEnvF5 | TCGTTGCAGACCCTTATCCCAG | 7832-7853 | CpzEnvR5 | AACAGCTATTGCAGTTGCAGAAGG | 8158-8181 | 326 |
| P | Ltr100-outF | AGCTGCATATAAGCAGCCGC | 9003-9022 | Ltr100-R | GGCAAGCAGRGAGCTGGAAA | 475-494 | 565 |
| Q | Ltr100-inF | CTTGTACTGGGTCTCTCT | 9029-9046 | Ltr100-R | GGCAAGCAGRGAGCTGGAAA | 475-494 | 485 |

1. See Fig.2 for position in SIVcpz-Gab4 genome
2. Nucleotide legend: R=A/G, Y=C/T, H=A/C/T, W=A/T, N=A/C/T/G
3. Size of fragment (in base pairs)

* The first two consensus primers pairs used
